# Supplementary material for: Utilising Hyperspectral Autofluorescence Imaging in the Objective Assessment of Disease State and Pain in Patients with Rheumatoid Arthritis
Source: Int J Mol Sci. 2024 Nov 8;25(22):11996. doi: 10.3390/ijms252211996 (PMC11593821; doi:10.3390/ijms252211996)
Supplement: Supplementary file 1 [file ijms-25-11996-s001.zip › ijms-3260845-supplementary.pdf]

Supplementary Material

Utilising Hyperspectral Autofluorescence Imaging in the Objective Assessment of Disease State and Pain in Patients with Rheumatoid Arthritis

Florence Lees [1,2], Saabah B. Mahbub [3,4], Martin E. Gosnell [5], Jared M. Campbell [3,4], Helen Weedon [6], Abbas Habibalahi [3,4], Ewa M. Goldys [3,4,*], Mihir D. Wechalekar [6], Mark R. Hutchinson [1,2] and Tania N. Crotti [1]

1 School of Biomedicine, University of Adelaide, Adelaide, SA 5005, Australia
2 ARC Centre for Excellence for Nanoscale Biophotonics, University of Adelaide, Adelaide, SA 5005, Australia
3 Graduate School of Biomedical Engineering, University of New South Wales (UNSW), Sydney, NSW 2052, Australia
4 ARC Centre of Excellence for Nanoscale Biophotonics, University of New South Wales (UNSW), Sydney, NSW 2052, Australia
5 Quantitative Pty Ltd., 118 Great Western Highway, Mount Victoria, NSW 2786, Australia;
6 Department of Rheumatology, Flinders Medical Centre, Bedford Park, SA 5042, Australia
* Correspondence: e.goldys@unsw.edu.au

Corresponding author: Prof Ewa Goldys

ORCID ID: 0000-0001-5116-2534,

Supplementary Methods

1. CD68 staining:

Sections were washed in 1x PBS (pH 7.4, 3 x 5 minutes) then incubated with blocking buffer (0.1% NaN3/1% H2O2/TPBS) for 20 minutes at room temperature. Following 1 x PBS wash, sections were blocked with 20% normal donkey serum for 30 minutes at room temperature. Sections were incubated with primary antibody (EBM11 1:400 Catalogue #M0718, Dako Australia), diluted in 1 x PBS/1% BSA overnight at 4°C and washed in 1 x PBS. Secondary goat anti-mouse antibody (HRP 1:100 Catalogue #P0447; Dako Australia) diluted in 1 x PBS 1% BSA/10% NHuS were incubated for 30 minutes at room temperature, followed by 1 x PBS wash. Sections were then incubated with tertiary rabbit anti-goat antibody (HRP 1:100 catalogue #P0449; Dako Australia) for 30 minutes at room temperature, followed by 1 x PBS wash. Sections were incubated with substrate chromogen (AEC ready-to-use #K3469; Dako Australia), counterstained with haematoxylin then mounted with Aquatek. CD68 positive cells were assessed within a 0.68 mm^2 region of interest (ROI) within the sub-lining by two blinded observers as per a previously published semi-quantitative scoring method [1].

2. Collagen II stain:

Sections were washed then incubated with blocking buffer for 60 minutes at room temperature. Primary antibody diluted in blocking buffer was added and incubated overnight at 4ºC and washed. Donkey anti-rabbit secondary antibody was diluted in 1 x PBS and added to sections before incubation for 60 minutes at room temperature and washed. The intensity of staining was measured using the software Fiji (Image J version 2.0).

The following reagents were used:

Blocking buffer: 1 x PBS10% normal horse serum/0.01%NaN3

Primary antibody: Anti-Collagen II 1:100 Catalogue # ab34712, Abcam

Secondary antibody: Drb 594 1:1000 catalogue # A212047; Molecular probe Life technology

Washing steps involved washing in 1x PBS (pH 7.4) 3 times for 5 minutes.

3. Preparation for imaging of Synovial tissue (ST):

ST was fresh frozen in optimal cutting temperature compound (Sakura) and stored in a -80°C freezer. 5 µm thick sections were cut using a cryostat and placed onto a coverslip. Tissue sections were heated at 37°C with 15 µl of Hanks Balance Salt Solution (HBSS) (catalogue #H6686, Sigma Aldrich) placed on top of the sample to prevent excess drying from causing cellular stress and having a confounding effect on HSI data.

4. HAI Data analysis:

The 69 fluorescent channels were produced by a multispectral excitation lamp (Quantitative™, AU) with five different epifluorescence filter cubes. Images were captured by an EMCCD (Nuvu™ Canada) (sensor size 1024×1024 pixels with an operating temperature of -60°C to reduce sensor-induced noise). The maximum image acquisition times in each channel were set from 0.2 to 2 seconds and, 3 to 5 images were used to allow the photon count for each channel to be averaged. This enabled the collection of higher quality images (higher signal-to-noise ratio for lower-powered LED) while minimising the photobleaching of the biological samples. For flattening images and background removal, two sets of reference images; comprising of imaging medium and calibration fluid (mixture of 30 µM NAD(P)H; catalogue #10107735001 and 18 µM FAD; catalogue #F6625; Sigma Aldrich) were used. The calibration fluid was additionally characterised on a fluorimeter (FluroMax 4 Plus c; Horiba, Japan). Graphic User Interface (GUI) software[2] was used for pre-processing, flattening, processing, and segmentation (with the help of BF images).

5. Compressing hyperspectral images to facilitate imaging of group signatures by using a deep learning autoencoder algorithm

A deep learning algorithm based on an autoencoder[3] was developed in order to create an expertly interpreted version of hyperspectral image datasets which highlight the group signatures obtained from synovial tissue. This algorithm compresses the highly dimensional hyperspectral images into exactly four colour (CMYK) variables enabling clear visualisation of regions where sample autofluorescence was modified by molecular changes within the considered groups. This procedure was applied to smooth and pre-processed hyperspectral images which were standardised by setting the mean of each variable to zero and the standard deviation to one. The variables values were then linearly transformed to fit into a range between -0.5 and 0.5. The overall technique is built on the analysis of a limited number of spectral features on an individual pixel's basis found to yield good group discrimination. These were

utilised to generate group masks. The pixel features used here are ratios of the following channel pairs (3/12, 3/20, 7/18).

The procedure has two phases (Phase 1 and Phase 2) as demonstrated in Supplementary Figure 1.

Phase 1: The phase 1 algorithm (Supplementary Figure 1 left) consists of an iterative conventional deep learning sparse autoencoder running in a loop. The autoencoder includes the encoder and decoder parts. Based on a linear transformation of selected feature values (intensities and ratios) using flexible coefficients ("weights"), the encoder converts the pixel feature values into a low dimensional (latent) space (four variables to make it possible to be visualized with 4 CMYK colours) at every iteration. In addition, the encoder integrates nonlinear activator functions to avoid the limitations of linear transformation for example, PCA. The decoder reproduces a prediction of the original feature image. To this end, the decoder maps back the CMYK colour images (four encoded variables) onto the same dimension of the input data (feature images), and it incorporates its specific linear activation functions. The mean square error was selected as the cost function criterion for the algorithm. At each iteration, the difference of the output image with the original image is evaluated based on cost function calculation, and a regularizer term reduces overfitting risk. The cost function is minimized by autoencoder through obtaining the best encoder and decoder weights which can regenerate the training data from low dimensional subspace (the four CMYK space)[4]. To train the network, a supervised algorithm that was found to be faster than standard backpropagation is used. A sparsification strategy (Sparse Autoencoder) was utilised where the sparse autoencoder cost function corrects hidden layer neuron activations as a training sample is introduced, which facilitates the formation of specialized features. After the training process is complete, the outcome CMYK image approximates the original image but in 4 dimensions each of which is exclusively informative. As an alternative to the original feature images obtained from all available spectral channels, Supplementary Table 2, this methodology enables colours to express specific features corresponding to the data and reduces the hyperspectral image dimensionality.

Phase 2: Phase 2 is designed to focus more on pain-related features and anatomy in our algorithm. To this end, the decoder is eliminated and replaced by a different supervised classification layer (Supplementary Figure 1 right) where each pixel is labelled based on image origin from a particular patient group (here Active RA and Inactive RA).

The new classifier layer and the encoder section of the pretrained autoencoder are trained again using cross entropy cost function which is a new criterion and scores similarity between the output and label. While the algorithm is iterating, the weights continue to be corrected to finetune four latent (CMYK) variables and obtain complex information associated with pain-discriminating.

Different cost criterion functions are used for Phase 1 and Phase 2 algorithms. Therefore, the mean square error is sacrificed in the second algorithm which was employed to optimize cross-entropy. However, this is suitable as the aim is to obtain tissue anatomy with enhanced discrimination power of pain-no pain groups, but with minimized variance.

The CMYK images this obtained are an optimized non-linear transformation of the original hyperspectral images and they display the tissue anatomy with a focus on RA Active and RA Inactive-discriminating information.

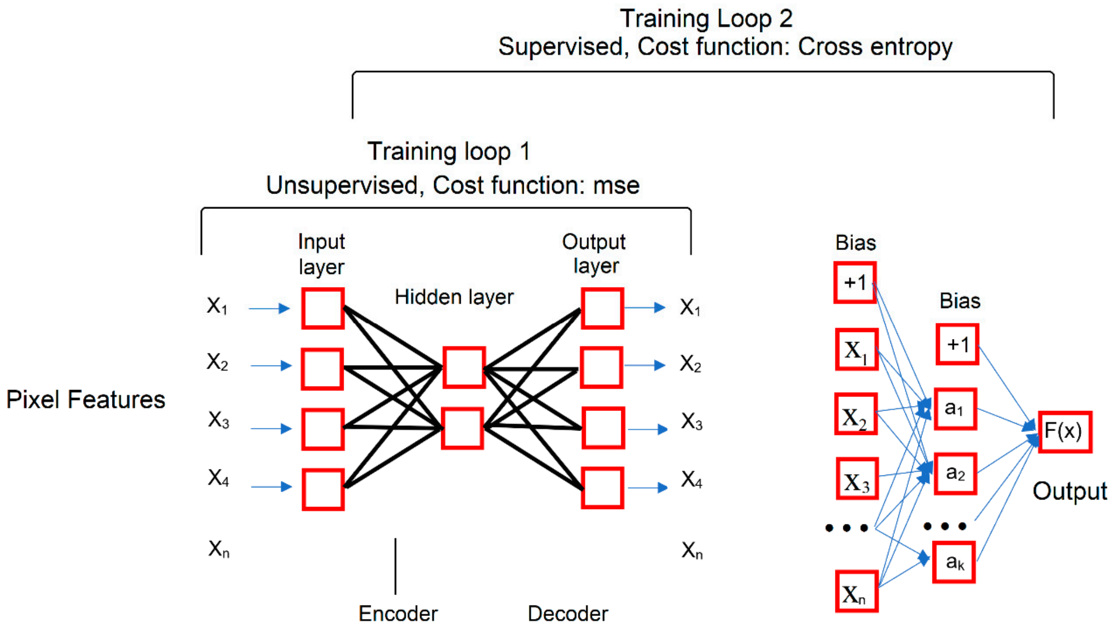

Supplementary Figure 1. Autodecoder schematic illustration to achieve the "RA Active-RA inactive" group projection. Phase 1: Transforms the pixel feature values to four (CMYK) variables using an unsupervised sparse autoencoder which indicates hyperspectral tissue anatomy. Phase 2: Replaces the decoder from Phase 1 by another training loop. The training process is conducted again to improve image anatomy agreement with patient group discrimination. The outcome is RA Active-RA Inactive"-discriminating anatomy images in the 4-dimensional (CMYK) space.

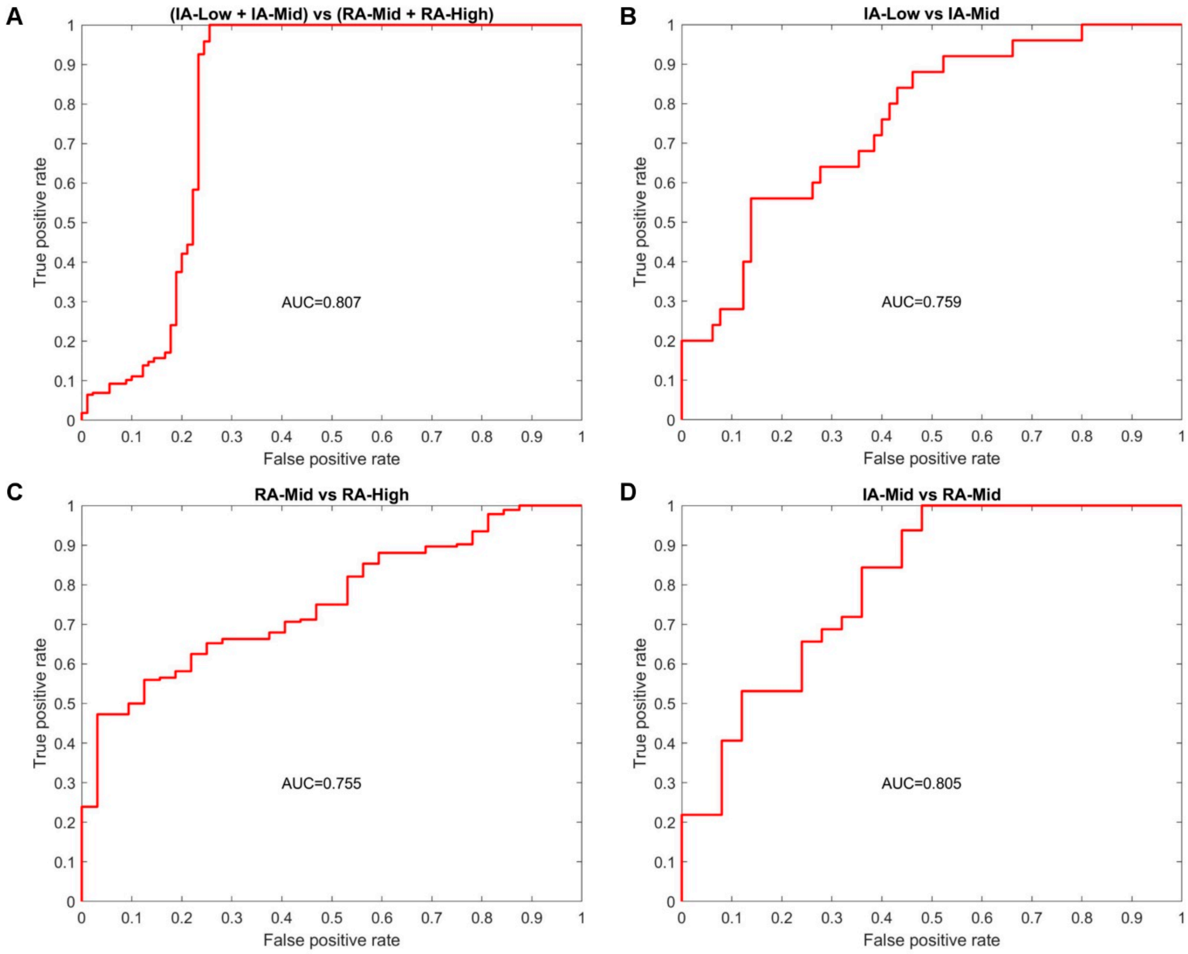

Supplementary Figure 2. AUC results for figure 3.

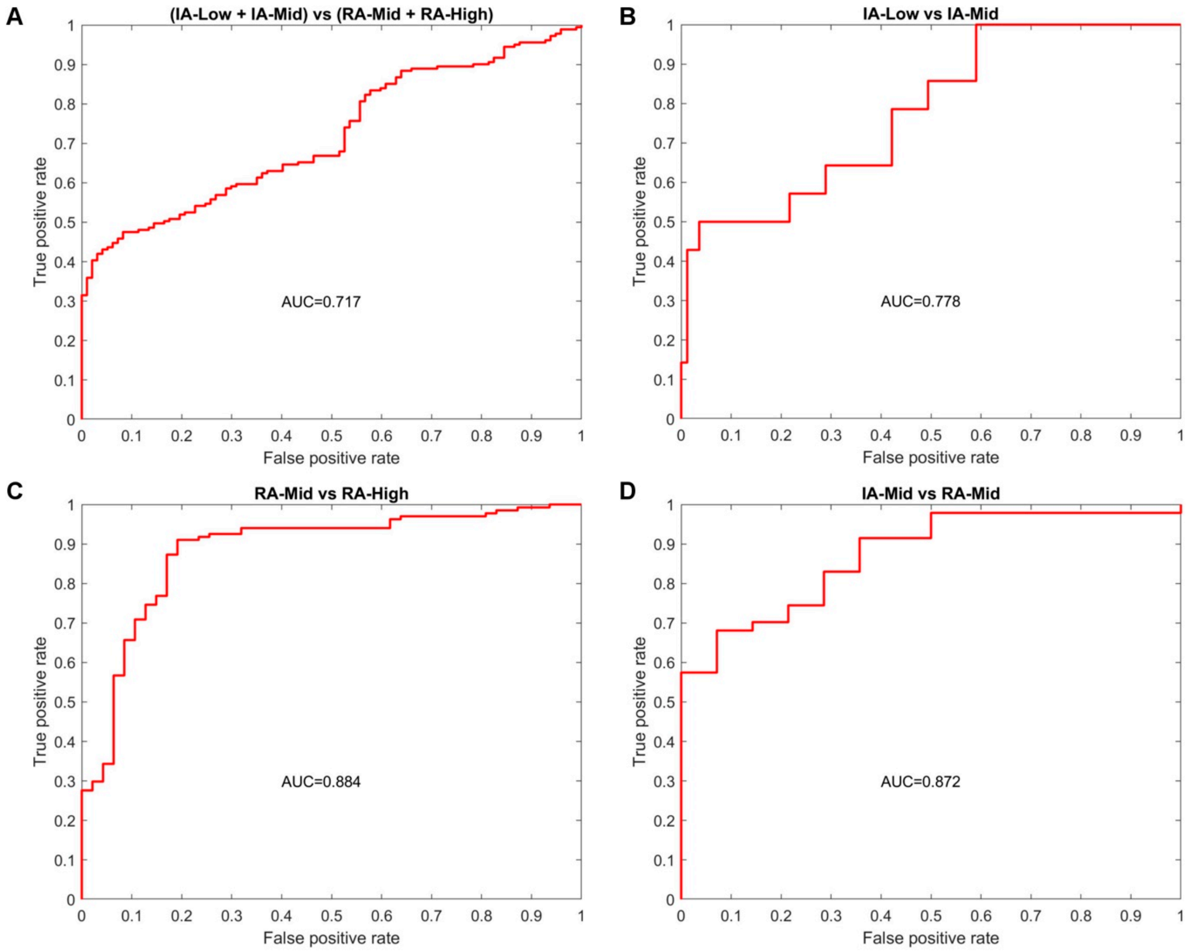

Supplementary figure 3: AUC results for figure 5

Supplementary table 1. Characteristics of patient samples outlining disease activity and treatment components, along with synovitis observed. 1A) outline of inactive patients, B) outline of active patients.

**Table 1A**								**DAS28**			
**Sample No.**	**VAS_Pain**	**DAS**	**Synovitis severity and details as documented on arthroscopy**	**VAS_Fatigue**	**HAQ**	**SEX**	**treatment**	**28TJC**	**28SJC**	**ESR**	**Pt Global**
AP43/13c	3	1.1	Mainly flat synovium but occasional villous projections, mainly fatty cores on medial wall of suprapatellar pouch	4.00	0.00	M	Abatacept	0.00	1.00	3.00	4.00
AP21/14f	2	2.3	Patchy mild synovitis present	89.00	0.13	F	Triple therapy	1.00	3.00	4.00	18.00
AP32/13c	9	2.2	Patchy synovitis present	7.00	0.88	M	Abatacept	0.00	0.00	20.00	6.00
AP07/15b	1	1.6	patchy minimal synovitis present	0.00	0.00	F	Triple therapy	0.00	0.00	10.00	1.00
AP48/14b	4	1.8	minimal patchy synovitis present	14.00	0.00	M	Abatacept	0.00	2.00	5.00	3.00
AP11/13b	4	1.3	Very patchy synovitis present, but generally fairly bland synovium	2.00	0.13	F	Triple therapy	4.00	2.00	51.00	42.00
AP51/13c	48	2.0	Patchy synovitis present	8.00	0.63	M	Triple therapy	2.00	0.00	5.00	8.00

AP27/12c	6	2.6	Florid synovitis throughout knee joint	4.00	1.38	F	Triple therapy	0.00	4.00	13.00	19.00
AP43/13B	4	1.6	Mainly flat synovium but occasional villous projections, mainly fatty cores on medial wall of suprapatellar pouch	4.00	0.00	M	Abatacept	0.00	1.00	3.00	4.00
**Mean**	**9.0**	**1.8**		**14.7**	**0.35**			**0.8**	**1.4**	**12.7**	**11.7**
**Median**	**4**	**1.8**		**4**	**0.13**			**0**	**1**	**5**	**6**
**Table 1B**											
**Sample No.**	**VAS_Pain**	**DAS**	**Synovitis severity and details as documented on arthroscopy**	**VAS_Fatigue**	**HAQ**	**SEX**	**treatment**	**28TJC**	**28SJC**	**ESR**	**Pt Global**
AP33/14f	49.00	6.9	Extensive synovitis present	49.00	1.13	M	Triple therapy	20.00	4.00	51.00	79.00
AP32/16e	26.00	4.5	Patchy, but severe synovitis present.	56.00	1.38	F	Triple therapy	2.00	4.00	26.00	64.00
AP54/14a	33.00	6.5	Moderate patchy synovitis present.	6.00	1.38	F	Adalimumab	15.00	13.00	81.00	17.00
AP32/12b	63.00	6.3	Florid synovitis present	32.00	1.75	F	Triple therapy	14.00	2.00	62.00	65.00
AP22/14c	94.00	7.1	extensive synovitis present	67.00	2.88	M	Abatacept	8.00	13.00	90.00	95.00
AP54/16b	76.00	7	Severe patchy synovitis present	74.00	2.63	F	Triple therapy	15.00	12.00	41.00	90.00

AP19/15c	65.00	4.8	Mild-moderate patchy synovitis present.	70.00	0.25	F	Adalimumab	7.00	4.00	20.00	45.00
AP110/11c	91.00	5.43	moderate-severe synovitis present throughout the joint	78.00	2.00	F	Triple therapy	12.00	7.00	10.00	81.00
AP41/12f	70.00	6.1	Florid synovitis present	69.00	1.38	M	Triple therapy	17.00	17.00	7.00	89.00
AP20/17b	72.00	8.1	very mild, patchy synovitis present	64.00	1.88	M	Adalimumab	23.00	14.00	115.00	72.00
AP34/13f	94.00	7.5	Patchy synovitis present	93.00	1.00	F	Adalimumab	22.00	22.00	26.00	94.00
AP105/11a	77.00	5.9	moderate-severe synovitis	56.00	1.05	F	Triple therapy	14.00	10.00	16.00	70.00
AP21/15F	42.00	4.8	significant knee synovitis present	65.00	0.75	F	Abatacept	4.00	5.00	39.00	34.00
**Mean**	**65.5**	**6.2**		**59.9**	**1.50**			**13.3**	**9.77**	**44.9**	**68.8**
**Median**	**70.0**	**6.3**		**65**	**1.38**			**14.00**	**10.0**	**39**	**72**

Supplementary Table 2:

Spectral Channels #	Excitation wavelength (bandwidth) (nm)	Emission wavelength (bandwidth) (nm)	Exposure (sec)	EM Gain	No of image accumulations to calculate "averages"
1	345	414	0.2	200	5
2	345	451	2	200	3
3	345	575	2	200	3
4	345	594	2	250	3
5	345	675	2	200	3
6	490	575	2	200	3
7	505	575	2	200	3
8	490	594	2	200	3
9	505	594	2	200	3
10	490	675	2	250	3
11	505	675	2	200	3
12	358	414	0.2	200	5
13	371	414	2	220	3
14	377	414	2	200	3
15	381	414	2	200	3
16	358	451	0.2	200	5
17	371	451	0.2	200	5
18	377	451	0.2	200	5
19	381	451	0.9	200	3
20	358	575	2	200	3
21	371	575	2	200	3
22	377	575	2	200	3
23	381	575	2	200	3
24	391	575	2	200	3
25	397	575	2	200	3
26	400	575	2	200	3
27	403	575	2	200	3
28	406	575	2	200	3
29	412	575	2	200	3
30	418	575	2	200	3
31	430	575	2	200	3
32	437	575	2	200	3
33	457	575	2	200	3
34	469	575	2	200	3
35	476	575	2	200	3
36	358	594	2	200	3
37	371	594	2	200	3
38	377	594	2	200	3
39	381	594	2	200	3
40	391	594	2	200	3

41	397	594	2	200	3
42	400	594	2	200	3
43	403	594	2	200	3
44	406	594	2	200	3
45	412	594	2	200	3
46	418	594	2	200	3
47	430	594	2	200	3
48	437	594	2	200	3
49	457	594	2	200	3
50	469	594	2	200	3
51	476	594	2	200	3
52	358	675	2	200	3
53	371	675	2	200	3
54	377	675	2	200	3
55	381	675	2	200	3
56	391	675	2	200	3
57	397	675	2	200	3
58	400	675	2	200	3
59	403	675	2	200	3
60	406	675	2	200	3
61	412	675	2	200	3
62	418	675	2	200	3
63	430	675	2	200	3
64	437	675	2	200	3
65	457	675	2	200	3
66	469	675	2	200	3
67	476	675	2	200	3
68	391	647	2	300	3
69	437	647	2	300	3

Supplementary Table 3: Spectral features used to differentiate segmented cells between inactive (*n*=90 cells) and active RA (*n*=216 cells) patients. Data are shown in Figure 3a.

No	Feature description
1.	Mean value of relative amount of NAD(P)H.
2	Mean value of relative amount of redox ratio.
3	Mean value of relative amount of flavins.
4	Mean value of relative amount of collagen.

Supplementary Table 4: Pain related spectral features used to differentiate segmented cells between IA-Low (*n*=65 cells) and IA-Mid (*n*=25 cells) patients. Data are shown in Figure 3b.

No	Feature description

1.	Mean value of relative amount of NAD(P)H.
2	Mean value of relative amount of flavins.
3	Mean value of relative amount of collagen.

Supplementary Table 5: Pain related spectral features used to differentiate segmented cells between RA-Low (*n*=32 cells) and RA-Mid (*n*=184 cells) patients. Data are shown in Figure 3c.

No	Feature description
1.	Mean value of relative amount of NAD(P)H.
2	Mean value of relative amount of flavins.
3	Mean value of relative amount of collagen.

Supplementary Table 6: Spectral features used to differentiate segmented cells between IA-Mid (*n*=25 cells) and RA-Mid (*n*=32 cells) patients. Data are shown in Figure 3d.

No	Feature description
1.	Mean value of relative amount of NAD(P)H.
2	Mean value of relative amount of redox ratio.
3	Mean value of relative amount of flavins.
4	Mean value of relative amount of collagen.

Supplementary Table 7: Spectral features used to differentiate segmented fibres between inactive (*n*=97 fibres) and active RA (*n*=181 fibres) patients. Data are shown in Figure 5a.

No	Feature description
1.	Mean value of relative amount of NAD(P)H.
2	Mean value of relative amount of redox ratio.
3	Mean value of relative amount of flavins.
4	Mean value of relative amount of collagen.

Supplementary Table 8: Pain related spectral features used to differentiate segmented fibres between IA-Low (*n*=83 fibres) and NA-Mid (*n*=14 fibres) patients. Data are shown in Figure 5c.

No	Feature description
1.	Mean value of relative amount of NAD(P)H.

2	Mean value of relative amount of flavins.
3	Mean value of relative amount of collagen.

Supplementary Table 9: Pain related spectral features used to differentiate segmented fibres between RA-Low (*n*=47 fibres) and RA-High (*n*=134 fibres) patients.

No	Feature description
1.	Mean value of relative amount of NAD(P)H.
2	Mean value of relative amount of flavins.
3	Mean value of relative amount of collagen.

Supplementary Table 10: Spectral features used to differentiate segmented fibres between non-active (*n*=14 fibres) and active RA *(n*=47 fibres) patients. Data is shown in Figure 5d.

No	Feature description
1.	Mean value of relative amount of NAD(P)H.
2	Mean value of relative amount of redox ratio.
3	Mean value of relative amount of flavins.
4	Mean value of relative amount of collagen.

Supplementary Table 11: Spectral features used for compressing the complex multidimensional hyperspectral image data into 4 colour (CMYK) images. (Representative images are shown in Figure 6).

No	Feature description
1	Ratio of mean value of top 10% of channel 3 and mean value of channel 3
2	Ratio of mean value of top 10% of channel 3 and mean value of top 10% of channel 12
3	Ratio of mean value of channel 3 and mean value of channel 12
4	Ratio of mean value of channel 3 and mean value of channel 20
5	Ratio of mean value of channel 7 and mean value of channel 18
6	Ratio of mean value of top 10% of channel 18 and mean value of channel 16
7	Ratio of mean value of top 10% of channel 12 and mean value of top 10% of channel 27
8	Ratio of mean value of channel 4 and mean value of channel 15

References

[1] Tak, P. P. *et al. Analysis of the synovial cell infiltrate in early rheumatoid synovial tissue in relation to local disease activity*. Arthritis Rheum. 1997;**40**(2):217-225.

[2] Mahbub SB, Tomczyk, Jakub and Goldys, Ewa M. *GUI_Preprocess version* 3.12 2020, github, DOI:10.5281/zenodo.4146738.

[3] Bengio, Y. *Learning deep architectures for AI.* (Now Publishers Inc, 2009).

[4] Møller, M. F. A scaled conjugate gradient algorithm for fast supervised learning. *Neural networks* **6**, 525-533 (1993).
